# Development of inferiority-compensation scale among high school students

**DOI:** 10.1186/s12909-022-03979-3

**Published:** 2023-01-12

**Authors:** Di Yang, Baiyang Qiu, Jin Jiang, Youkui Xia, Lingxiao Li, Yanting Li, Longli Luo, Xiaocui Liu, Jing Meng

**Affiliations:** 1 Key Laboratory of Applied Psychology, Chongqing Normal University, Chongqing, China; 2 Research Center for Brain and Cognitive Science, Chongqing Normal University, Chongqing, 401331 China; 3 Chongqing Youth Vocational & Technical College, Chongqing Youth Vocational & Technical College, Chongqing, China

**Keywords:** School students, Inferiority-compensation, Scale development, Chinese, Self-compensation, Others- compensation

## Abstract

An increasing number of high school students are inflicted by different degrees of mental disorders in learning, such as moodiness, learning difficulties, test anxiety, difficulty coping with frustration, etc., which are one of the factors leading to the inferiority of students. In the present study, the initial scale of inferiority compensation for high school students was developed through literature searching, expert evaluation, interviews, and an open scale. 1187 high school students were tested in different periods, after deleting an invalid 83 scales, including 461 copies of valid scale of exploratory factor analysis in the first stage and 643 copies of valid scale of confirmatory factor analysis in the second stage. The results showed that the inferiority compensation scale for high school students consisted of two sub-scale: self-compensation and others-compensation, ach two were composed of five dimensions including academic performance, physical fitness, social communication, appearance, and self-esteem. Confirmatory factor analysis showed that the total scale and the two sub-scale all had good structural validity (RMSEA≤0.08; CFI&IFI ≥ 0.9), and the combined reliability and values (such as the correlation coefficient of each dimension) of the two sub-scale were within the ideal range. With good reliability and validity (Cronbacα&KMO ≥ 0.90), and meeting the requirements of psychometrics, the scale can be used in the relevant research and practice of inferiority compensation for high school students.

**Supplementary Information:**

The online version contains supplementary material available at 10.1186/s12909-022-03979-3.

## Introduction

As Adler described in his book “What life should mean to you” [1], each of us in life has varying degrees of inferiority and since self-inferiority can make us nervous, depressed, and anxious, we will strive to obtain a sense of superiority in action to compensate for our inferiority and change its situation. In other words, everyone always believes that the situation where they are in needs to be improved at all times [2, 3].

The concept of compensation originally referred to the phenomenon of an individual causing a defective organ of the body to perform better than a perfectly normal organ [4, 5]. Now it gradually develops into a phenomenon where individuals experience frustration in the pursuit of a goal, or due to a physical deficiency and try to compensate with their strengths. Adler first extended the word compensation from physiology to psychology. Previous studies believed that everyone would make up for the self-inferiority caused by the insufficiency in reality or imagination to overcome this inferiority and become a superior person to others [6, 7]. Thus, the current interpretation of inferiority compensation refers to a series of phenomena that which behavioral and cognitive efforts made by individuals enable the individual abilities to be improved in some aspects and function reflected overcome inferiority to regain self-confidence. The method of compensation is to make up for their shortcomings and inferiority through efforts and achievements in some aspects, to give full play to the individual subjective initiative [8], and to use and transform the objective environment to adapt to their problems in society [9].

From the perspective of the executors of compensation behavior, Adler believes that the perpetrator of compensatory behavior is only the self and no others, i.e., there is only self-compensation [10]. Some scholars who support Adler’s view also believe that inferiority is unique to individuals, independent of others [11, 12], and others do not replace it [13–15]. Thus, from this point of view, these scholars believe that compensation can only be completed by individuals with inferiority, that is, there is only self-compensation without compensation for others.

However, due to the diverse self-concepts between the eastern and western cultural backgrounds [16], the ego from oriental collectivism includes the perception of others, while western individualism, where members advocate personal values and are used to distinguishing themselves from the outside, does not include anyone else [17]. Hence, some scholars put forward different opinions that the individual’s sense of insecurity, and feelings of powerlessness and disappointment due to the inability to achieve the goal, can be regarded as an inferiority complex [18]. In real life, this inferiority complex whether from physical, psychological, or social obstacles, can all be compensated. In addition, the compensation behavior does not necessarily have to be completed by the inferiority of self, it can also be completed by others or groups, even by the whole country or nation [19, 20]. Friehe et al. indicate that if a person incorporates the object of comparison into his system, the threat of others’ success will be significantly reduced [13]. A study by Booysen et al. indicates that there is more possibility to occur others-compensation in individuals under a collectivist culture than under an individualist culture [21]. In addition, some scholars proposed compensation for others from the perspective of the compensation behavior executor and further explained that compensation for others is an objective completing method different from self-compensation [6, 17, 22]. Since Adler considers that the individual obtains superiority in the form of social identity, it is undeniable that there are other forms of identity to gain excellency, which can be seen as a substitute compensation obtained through the identity of others.

In the development of his compensation theory, Adler insisted that is not a symbol of deformity, but a normal phenomenon in the personal pursuit of excellence [1]. And inferiority has a positive or negative effect on individual development depending on the compensation attitude of the individual [13, 23]. If an individual manages to make up for the defects of his organs or abilities, he will improve the environment around him by direct and practical means, and in the strength of his internal beliefs, and consequently transform the inferiority at this time into the internal motivation for progress. Then compensation is effective, and inferiority plays a positive role.

If a person simply pursues the sense of superiority but does not attempt to change the surrounding environment, then compensation will point to the useless side of life, make himself intoxicated or numb in fantasy, and obtain the emotional experience of self-confidence in the way of self-paralysis. In this case, the inner self-abasement will not disappear [24]. Adler believes that such compensation will only become a tumor of their psychological growth, and eventually form an inferiority complex [1, 2]. But inferiority complex will make people drift apart from society, discouraged, and lack self-confidence, and people with an inferiority complex do not believe that they will make any progress in life [12, 25].

Psychological problems prevail among high school students [26]. If they can actively make up for the negative factors, they will gain confidence, have a higher level of self-evaluation, and their inferiority will be improved to some extent [27]. However, if students do not actively make up for their shortcomings while choosing to escape, the inferiority complex gradually forms, resulting in psychological abnormalities. For that reason, for high school students, inferiority compensation is one of the vital approaches to getting rid of inferiority, gaining self-confidence, and developmental health.

Yet, based on cultural background differences, the inferiority compensation scale has not been compiled by scholars. As mentioned above, mental problems are prevalent issues among high school students. It remains unclear how they view their inferiority, why avoid talking about the topic of inferiority, and how to enhance their social adaptability and self-confidence. Here, we hope to answer the questions above by investigating the patterns of inferior compensation for high school students.

## Materials and methods

There were 2 main phases to the study. In the first development phase, potential question items were generated and tested in face-to-face interviews and 2 pilot rounds. In the second validation phase (the main study), we confirmed the internal reliability of the scale and examined construct validity and test-retest reliability.

### Participants

#### Predictive samples

Convenience sampling was applied to obtain representative samples of school students from high schools in Chongqing, China. Participants were chosen from high school students rather than primary students because high school students could better understand the content on our scale. Students filled out the scale on the spot-on laptops or online in the first round of the scale. Altogether 500 scales were distributed. After screening according to the inclusion and exclusion criteria and eliminating the ineligible scale, a total of 461 predictive scales were collected and the scale return rate was 92.2%. The survey time was from January 2020 to March 2020.

Convenience sampling was applied to obtain samples of school students. School students from high schools in Chongqing, China were recruited in the second-round study to fill out the scale on the spot (these students did not participate in the first round of the scale). Altogether 687 scales were distributed. After screening according to the inclusion and exclusion criteria and eliminating the ineligible scale, a total of 643 predictive scales were collected and the scale return rate was 93.59%. The second survey time was from October 2020 to March 2021.

#### Scale development

Literature retrieval [28–30]. We retrieved the relevant literature at home and abroad, where we selected, analyzed, and collated the phrases and sentences that can reflect the self-inferiority compensation of high school students.

We first identified relevant conceptual dimensions of inferiority-compensation and developed a pool of 25 items based on a review of the literature and consultation with experts, and in-country stakeholders.

#### Candidate question items and face-to-face interviews

Using a structured interview, investigators identified 4 open-ended schemes and concepts regarding the participants the methods that can be used to overcome inferiority, and how the honors of others have an impact on overcoming inferiority.

We also distributed 156 copies of the scale, and, as a result, 105 of them were collected effectively, and the recovery rate was 67.3% (some students were sensitive to the word inferiority, so they did not fill out the scale). The investigation time was November 2019.

After eliminating the equivocal, irrelevant, and unmanageable answers, the obtained content was sorted out with the relevant literature, then discussed with the professors of psychology in related fields. At last, we divided the inferiority compensation scale into two sub-scale: the self-compensation sub-scale and the others-compensation sub-scale. The dimensions of the sub-scale are obtained through the open scale results of interviews and the five dimensions of the inferiority scale revised by Chavez and Fritz [31, 32]. And Final dimensions identified included: academic performance, physical fitness, social communication, appearance, and self-esteem. We drafted about 4–6 items for each of these conceptual domains. Part items were adapted from Hirao’s job inferiority scale [22] as shown in Table 1.

**Table 1:** List of open-ended scale results

	Frequency	Operational Definition	Dimensions	Explanation
self-compensation	76	Some behavioral phenomena in which individuals take the initiative to overcome their shortcomings and exploit their strengths in order to improve their abilities in a certain area	academic performance	Proactively compensate for for academic shortcomings
	25		physical- fitness	Proactively compensate for physical deficiencies
	53		social communication	Proactively compensate for social deficiencies
	14		appearance	Proactively compensate for lack of appearance
	43		self-esteem	Proactively compensate for self-esteem deficiencies
others-compensation	45	Individuals feel a series of self-confidence boosting phenomena indirectly brought about by the success or honor of their team, collective and relational others	academic performance	Glory of others boosts individual academic confidence
	20		physical- fitness	Glory of others enhances individual physical self-confidence
	39		social communication	“Glory to others increases social confidence
	9		appearance	“Glory to others increases one’s self-confidence in appearance
	17		self-esteem	“Glory to others increases self-esteem and self-confidence

The set of items was introduced by “Because one subject is poor, I will make up for it by taking advantage of other subjects?”. Following items were the statements like “Because of the weak reading ability, I will make up by reading extracurricular books”, “Because of the weak sports scores, I make up for the promotion by attending sports meetings” and “I praise myself in front of the opposite sex to be looked up to.” Response options included completely inconsistent, basically inconsistent, uncertain basically consistent, completely consistent. The detailed items are shown in Additional file 1 Appendix 1.

#### Assessment of validity and reliability

To assess the concurrent validity of the inferiority-compensation scale (ICS), the compensation scale [33] was used as a criterion measure. There are 13 items on the scale. The Cronbach α coefficient of the internal consistency of the scale was 0.833, and the split-half reliability was 0.734. In the current study, the Cronbach α coefficient of the scale is 0.809, and the test-retest reliability after one month is 0.793.

#### Data management and statistical analysis

Data were double-entered and cross-checked using the statistical software Epita3.1. An exploratory factor analysis was conducted on the data collected in Phase 1 using SPSS22.0. Validation factor analysis was conducted on the data collected in Phase 2 using Amos 23.0.

## Results

### Item analysis

We calculated the critical value (around 27%). According to independent samples, and t-tests, items with a nonsignificant difference were removed. The statistical analysis results showed the self-compensation sub-scale of the inferiority compensation scale finally includes 33 items; and the others-compensation of the sub-scale inferiority compensation scale finally includes 28 items.

The correlation between the total scores and items scores: in order to investigate the correlation between the total scores and the scores of 33 items in the self-compensation sub-scale and 28 items in the others-compensation sub-scale, Pearson test was used to analyze the correlation between each item score and the total scores in the present study, and the items were deleted according to the correlation coefficient of 0.4. The results showed that the self-compensation prediction scale left 31 items in the end, and the others-compensation prediction scale finally left 27 items.

### Construct validity

The KMOs for the self-compensation and other-compensation subscale in this study were 0.92 and 0.91 respectively, with p less than 0.001, which supported that the data were appropriate for exploratory factor analysis.

The exploratory factor analysis was analyzed by the main component analysis method and the maximum variance method [34], and the criteria were as follows: (1) the items with a load less than 0.4 were deleted, and the commonality of the items was greater than 0.2; (2) the load of multiple factors was not large, and the maximum two load differences were less than 0.2; (3) the items that could not be explained or were obviously improperly classified, and the factors included less than 3 items. Using the principal component dimension extraction analysis method, and the promax oblique rotation method, additionally combing with the screen test with eigenvalues greater than 1, the two sub-scale finally extracted five factors, which were consistent with the inferiority compensation theory. As a result, we deleted 10 items in the self-compensation sub-scale, retained 21 items, and the total variance of explanation was 58.97%. On the other hand, we deleted 6 items in the others-compensation and retained 20 items., and the total variance of explanation was 57.85%. The detailed data of factor analysis is shown in the Tables 2 and 3.

**Table 2:** Rotation matrix of the self-compensation subscale

Item	Factor Loading					Communality
	1	2	3	4	5	
A8	0.867					0.740
A9	0.754					0.701
A12	0.572					0.558
A32		0.802				0.506
A33		0.802				0.608
A34		0.695				0.712
A31		0.552				0.550
A35		0.542				0.553
A2			0.858			0.596
A3			0.782			0.695
A1			0.747			0.635
A4			0.577			0.513
A6			0.559			0.483
A26				0.702		0.578
A29				0.662		0.533
A28				0.560		0.628
A15					0.885	0.583
A14					0.689	0.719
A20					0.483	0.516
A16					0.428	0.513
A18					0.400	0.464
Initial Eigenvalues	7.353	1.568	1.367	1.087	1.010	
Accumulative Contribution Rate	35.014	42.481	48.991	54.167	58.974

**Table 3:** Rotation matrix of the others-compensation subscale

Item	Factor Loading					Communality
	1	2	3	4	5	
B2	0.869					0.718
B3	0.842					0.685
B1	0.750					0.611
B4	0.533					0.494
B7		0.885				0.727
B8		0.822				0.740
B11		0.628				0.584
B14			0.860			0.646
B13			0.648			0.552
B17			0.592			0.496
B16			0.581			0.521
B12			0.574			0.549
B15			0.443			0.489
B22				0.776		0.624
B24				0.610		0.577
B23				0.550		0.562
B28					0.822	0.637
B29					0.793	0.726
B30					0.681	0.513
B27					0.557	0.515
Initial Eigenvalues	6.469	1.526	1.449	1.123	1.003	
Accumulative Contribution Rate	32.344	39.975	47.222	52.838	57.850	

### Confirmatory factor analysis

In order to ensure that the structure of the total high school students’ inferiority compensation scale conforms to the measurement standard, confirmatory factor analysis is used to fit the structure of exploratory factor analysis. According to the exploratory factor analysis of the fitting degree test, we analyzed the correctness of its results and the reliability of the theoretical model. Amos 23.0 was used for confirmatory factor analysis of 643 copies of the scale. According to the model fitting index, the structure models of the two sub-scale and the total self-inferiority compensation scale are verified.

**Fig. 1:**
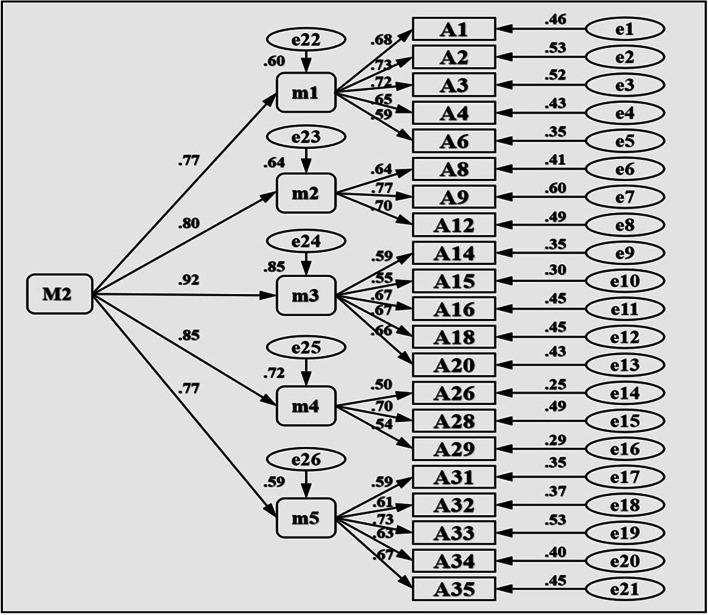
Self-compensation second-order model

**Fig. 2:**
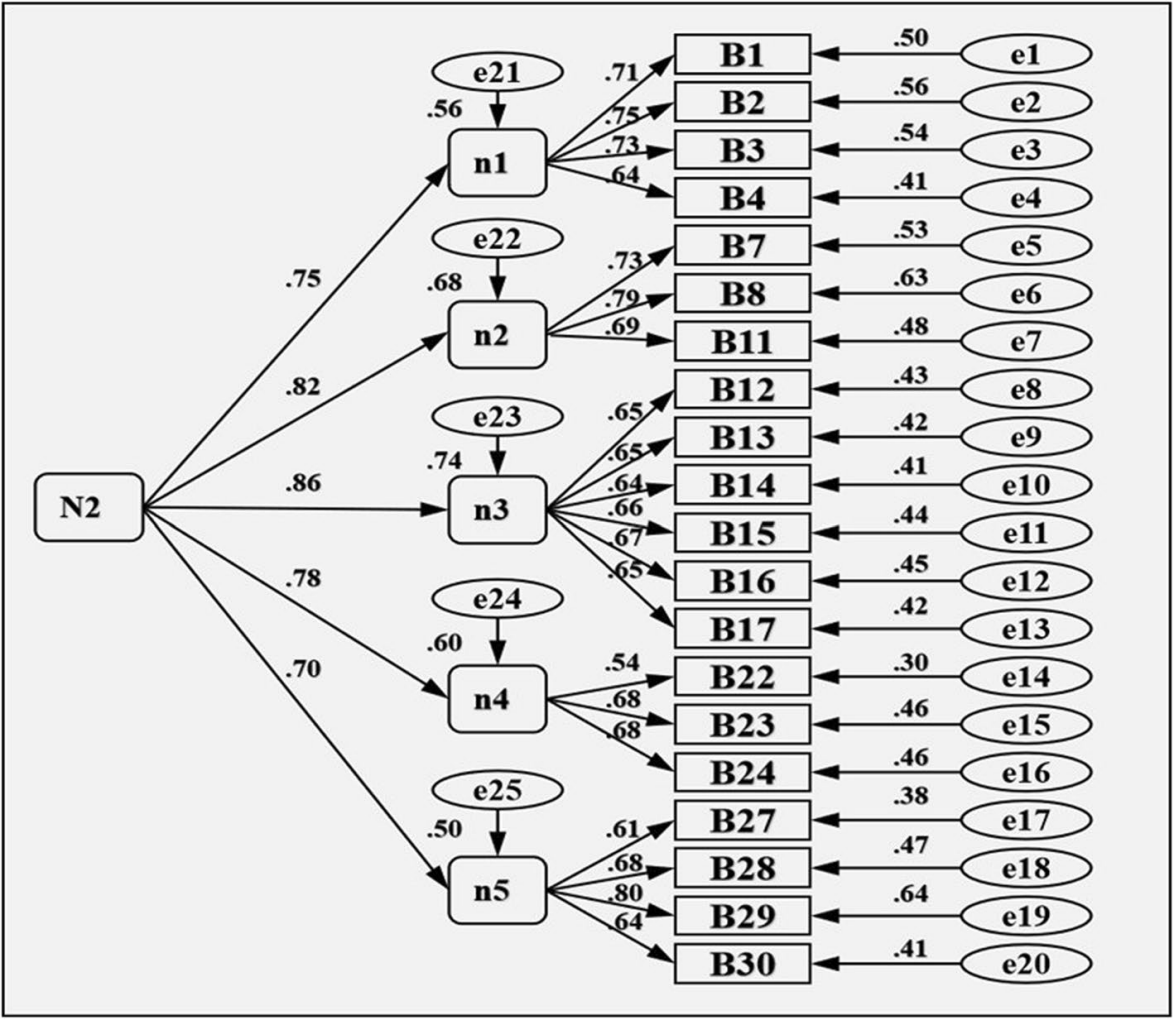
Others-compensation second-order model

When evaluating the structural rationality of a model through confirmatory factors, there are generally three types of indicators [15]: (1) Alternative test index. *χ*^*2*^ (chi-square) test, generally using *χ*^*2*^*/df*, that is, the ratio of chi-square value to degrees of freedom as an indicator, the theoretical expectation value is 1, in practical research, less than about 5 is considered a good model. (2) Absolute index. A commonly used metric is the “root mean square of approximate error (RMSEA)”. It is generally believed that RMSEA< 0.05 is a good fit, RMSEA between 0.05 ~ 0.08 is an acceptable fit, and RMSEA≥0.10 is a poor fit. In short, RMSEA less than or equal to 0.08 is acceptable, the smaller the better. (3) Relative index. Commonly used indicators are “increasing fitting index (IFI)”, “comparative fitting index (CFI)”, etc., the value is between 0 ~ 1, the closer 1 model fit, the better, generally required to be above 0.90. Based on Marsh’s research, in the first-order model [35], if the lowest correlation between the dimensions is greater than 0.50 (the lowest correlation between the two subscales in this study is 0.51, *P* < 0.001), further attempts can be made to explore the existence of the second-order model. Results In terms of overall fit, the overall fit of the second-order model and the first-order model was good, and according to the principle of simplicity, the second-order model was more simplified than the first-order model. The model under the first-order dimension represents the goodness-of-fit related indicators between the items (test questions) and the five objects, namely, self-esteem, social interaction, academic performance, physical fitness, and appearance. Here, M1 and N1 were used to separately represent the models of the sub-scale of self-compensation and others-compensation. The model under the second dimension represents the fitting degree parameters between self-compensation and others-compensation and those five dimensions and items, respectively. Here, M2 and N2 were used to represent the models of self-compensation and others-compensation. In addition, by fitting all the question models of the total scale, the RMSEA was greater than 0.1, but by calculating the average value of each dimension and then fitting again [36], the RMSEA is 0.8 to reach the ideal range, therefore, here is the average value of each dimension. In this study, m1, m2, m3, m4, m5; n1, n2, n3, n4, and n5 represent the mean values of academic performance under the sub-scale of self-compensation and others-compensation, respectively. According to Table 4, Figs. 1 and 2 (A more minimalist second-order model is presented), the construct validity of the general scale and the two sub-scale is good.

**Table 4:** Indicators of validation factor model fit

	*χ*^*2*^	*df*	*χ*^*2*^*/df*	*CFI*	*IFI*	*RMSEA*
*M1* (self-compensation first-order model)	623.96	179	3.48	0.90	0.90	0.06
*M2* (self-compensation second-order model)	637.43	184	3.46	0.90	0.90	0.06
*N1* (others-compensation first-order model)	526.28	160	3.28	0.92	0.92	0.05
*N2* (others-compensation second-order model)	569.67	165	3.45	0.91	0.91	0.06
Total scale	168.37	34	5.48	0.93	0.93	0.08

### Reliability

In this study, Cronbach’s coefficient was used to measure the reliability of the scale. As shown in Table 5, in terms of internal consistency reliability, Cronbach’s α for the reliability analysis of the self-compensation subscale was good, at α = 0.90. In terms of the five factors, Cronbach’s α was 0.80 for academic performance, 0.74 for physical fitness, 0.76 for social communication, 0.68 for appearance, and 0.77 for self-esteem. These results demonstrated that the items were internally consistent.

**Table 5:** Reliability analysis of the self-compensation subscale

Dimensions	Academic performance	Physical-fitness	Social communication	Appearance	Self-esteem	Total
Cronbach α	0.80	0.74	0.76	0.68	0.77	0.90
Split-half reliability	0.73	0.70	0.71	0.67	0.72	0.83

As shown in Table 6, in terms of internal consistency reliability, Cronbach’s α for the reliability analysis of the others-compensation subscale was good, at α = 0.90. In terms of the five factors, Cronbach’s α was 0.79 for academic performance, 0.77 for physical fitness, 0.81 for social communication, 0.66 for appearance, and 0.77 for self-esteem. These results demonstrated that the items were internally consistent.

**Table 6:** Reliability analysis of the others-compensation subscale

Dimensions	Academic performance	Physical-fitness	Social communication	Appearance	Self-esteem	Total
Cronbach α	0.79	0.77	0.81	0.66	0.77	0.90
Split-half reliability	0.76	0.72	0.74	0.66	0.76	0.83

### Concurrent validity

In this study, the high school students’ inferiority compensation scale has two composite scales, so we can test its combination reliability. The combined reliability was calculated by the formula [37]. The combination reliability of self-compensation and others-compensation sub-scale was 0.67 ~ 0.80 and 0.70 ~ 0.80, respectively, which were both greater than 0.6 suggesting that the internal consistency of questions in each dimension was good in this study. The results of item reliability are within the acceptable range of indicators, indicating that each item has a good interpretation of the dimensions.

Construct validity can indicate the degree to which a test can detect a theoretical construct. In the present study, Pearson product-moment correlation was used to test the correlation between the total score of the formal scale and the scores of each dimension and to investigate the construct validity of the scale. According to Tables 7, 8 and 9, the correlation coefficients between the total scores of the two subscales and the scores of each dimension were 0.68 ~ 0.84 and 0.69 ~ 0.83, and the correlation coefficients between the scores of each dimension were 0.42 ~ 0.56 and 0.41 ~ 0.62, indicating that the dimensions were highly correlated with the scale, while the two dimensions were moderately low correlated. Furthermore, those results indicated that the two dimensions were independent and interrelated.

**Table 7:** Pearson’s correlation analysis of the dimensions of self-compensation with the total score of the subscale

Dimensions	Total	Academic performance	Physical-fitness	Social communication	Appearance	Self-esteem
Total	1					
Academic performance	0.77	1				
Physical-fitness	0.73	0.51	1			
Social communication	0.84	0.53	0.55	1		
Appearance	0.68	0.47	0.45	0.51	1	
Self-esteem	0.74	0.48	0.42	0.56	0.43	1

**Table 8:** Pearson correlation analysis of the dimensions of others-compensation with the total score of the subscale

Dimensions	Total	Academic performance	Physical-fitness	Social communication	Appearance	Self-esteem
Total	1					
Academic performance	0.74	1				
Physical-fitness	0.76	0.51	1			
Social communication	0.83	0.47	0.62	1		
Appearance	0.69	0.49	0.41	0.46	1	
Self-esteem	0.70	0.44	0.41	0.51	0.44	1

**Table 9:** Pearson correlation analysis of the total scale

Dimensions	Total scale	Others-compensation	Self-compensation
Total scale	1		
Self-compensation	0.94	1	0
Others-compensation	0.92	0.74	1

## Discussion

Adler’s theory of inferiority compensation is one of the core theories in the field of individual psychology. Each stage of the development of Adler’s inferiority compensation also reflects different characteristics, and when he perfected his theory, he absorbed and borrowed the research results of other scholars, roughly forming three more obvious stages.

In the first stage, Adler extended sex to the biology of the whole organism in his paper “A Study of Organ Defects and Psychological Compensation” [1]. He believed that the individual was a complete organism, so at that time he thought that compensation was only compensation given to defective organs.

In the second phase, Adler realized that inferiority was no longer limited to the body, but also existed in his own psychological and social disorders [38]. At this point, compensation is directed to the individual’s real or imagined state of life.

In the third stage, he integrates the individual with society, emphasizing that inferiority is a reflection of the individual’s lack of social value. In this regard, compensation is aimed at a social life, emphasizing the value of the individual’s contribution to society. Strive for the happiness of society to make up for the lack of personal social value [39].

Therefore, since the eighties of the last century, although inferiority has long become a hot spot in the field of personality, scholars have begun to pay attention to the problems related to inferiority and mental health, but as an important factor affecting personality, the compensation of its evolution has not been paid attention to. Domestic research on inferiority compensation is mainly limited to educators’ speculative summary of their own experience, and many reference suggestions for compensation are put forward from the perspective of overview, but there is a lack of empirical research.

In this study, the framework of our work research is rough as follows: Discussion and Definition of the construct → Expert views → ltem generation → ltems reduction (EFA) → ltems confirmation (CFA) → Reliability and Validity [40, 41].

We developed and validated the ICS for measuring the way to compensate for self-inferiority among middle and high school students. Contained two sub-scale and ten dimensions. The final version of the 41-item ICS contains two subscales – self-compensation, and others-compensation. Our results indicate that the ICS has acceptable validity and reliability for tracking and evaluating ways to compensate for self-inferiority.

First of all, to clarify the specific elements of inferiority compensation among middle and high school students, this study carried out an open scale survey, combed and analyzed the existing relevant studies, and constructed the theoretical structure of inferiority compensation by integrating the discussions on Adler’s inferiority and transcendence theory in previous literature.

At the same time, after repeated discussions with experts, professors, and graduate students of psychology in relevant research fields, the initial scale items were repeatedly screened by eliminating those questions of ambiguity. Therefore, the scale had good content validity. Secondly, the applicability of the questions was investigated through item analysis and related methods, and after the inappropriate problems were eliminated, there came the next round of analysis. Again, through exploratory factor analysis to get the final items, including the two sub-scale self-compensation and others-compensation.

This proves our previous assumption; Adler’s theory of inferiority compensation can exist in a cross-cultural context in ways of others-compensation.

Then, in the part of confirmatory factor analysis, based on the investigation results of each fitting index, χ2 / df, RMSEA, CFI, and IFI were all within the standard value range [37, 42], indicating that the scale has good model fitting. At the same time, the combined reliability value was greater than 0.6, which indicated that the reliability results of the items were within the acceptable range of indicators, and denoted that the items of the two sub-scale explained the dimensions well. Furthermore, convergence validity test results were all greater than 0.4, demonstrating that the facets of the two dimensions also had good explanatory power [43]. Finally, the correlations between the total scores of the sub-scale and the general scale, and the dimension scores were tested. As a result, the values were within the standard range, which further proved that the structural validity of the total scale was good. Finally, the Cronbach’s coefficient test showed that the reliability of the sub-scale and the general scale was good.

In summary, the theoretical structure of the Inferiority Compensation Scale for high school students is reasonable and has good reliability and validity. It can be used as a reliable measurement tool for follow-up studies of high school students’ inferiority compensation.

### Insights and limitations

It is a common phenomenon that an inferiority complex is prevailing among high school students, and in view of the general social phenomenon of only emphasizing test scores, people tend to pay little attention to the student’s mental health. Some data showed that about 1 / 5 high school students have different degrees of psychological disorders, such as repeated moodiness, lack of self-confidence, study difficulty, test anxiety, lack of concentration, large fluctuations in grades, and difficulty in coping with setbacks, which are one of the factors leading to students’ inferiority. Studies have shown that there is no significant difference between high school students’ sense of inferiority and senior high school students’ sense of inferiority [22, 27, 43]. In particular, it is difficult for high school students to avoid the comparison of academic performance, family background, and others in schools. If they find that others have what they want but do not possess, they will easily have mixed complex emotional experiences, such as inferiority, hostility, and resentment. But Maria and Cristiano also pointed out that part of the reason why a person can succeed is to compare with others to find their own shortcomings, which can encourage them to change themselves [20]. Hence, for example, in the performance ranking, students are inevitably compared with their classmates around them at school. How do they view this matter? If the comparison has already happened, and how would they deal with it, so it is necessary to pay attention to the psychological process of students, guide them to reasonably compare everything around them, and correctly view the results.

### Education recommendations

1. Pay attention to students’ inferiority complex, develop students’ consciousness of inferiority compensation, and improve students’ ability to adjust emotions when facing setbacks or failures. Teachers need to do their utmost to guide students to control their current negative emotions, and embrace life with positive optimism and smiles, so that students can have good self-regulation ability in the middle school stage, and can face and confront setbacks or failures with a positive and optimistic attitude. For example, educators should understand the pain that the students are experiencing at the moment, at the bottom of their hearts, and express understanding and sympathy for students. Such kind of empathy experience and accompany usually can give students great comfort and strength and it is a critical step for cheering students up to overcome their self-abasement, to guide students to well control the expression of emotions.
2. Strengthen the publicity of the positive role of inferiority. In the eyes of students, inferiority is generally a word that tends to be negative. Inferiority can make people feel faceless and unpopular, which also explains the phenomenon mentioned at the beginning that students are very evasive about the topic of inferiority. Yet, Adler said, in life, we more or less have different degrees of self-abasement, whether males or females, from the city or rural, and so on. Thus, to guide students to face up to their inferiority and strive to surpass themselves, teachers need to correct students’ inferiority bias in their learning and life. If self-abased students can often actively suggest themselves, and unaffectedly make friends with their self-abasement, then when students have reasonable beliefs, they can also face and solve problems at ease when encountering similar setbacks.
3. Attach importance to the environment around students to compensate for students’ inferiority. The effects of others-compensation and self-compensation are equally important. Whether it comes from the success of schoolmates, relatives, friends, or others, teachers need to strengthen the guidance of students’ jealousy and convert it into the driving force of students to complete self-improvement. If the success of others is regarded as an excellent example of power rather than regarded as a threat by students, it will have a multiplier effect on encouraging a student to improve their self-confidence to confront others who seem like or is really better than them. However, without the proper guidance of students’ psychological control in the face of others’ success, it is likely to lead to students’ serious jealousy, from month to month, and even lead to more serious inferiority. Certainly, in learning life, educators also should guide students not too much to show off their success in order to avoid making other students jealous. If the educator’s guidance is inappropriate, for example, when the individual is compared with the excellent, it is often the result that the self-inferiority will increase; When compared with losers, a weakening of inferiority is felt; This effect is especially pronounced if the object of comparison is similar to itself but cannot be surpassed [44].

In summary, the practical significance of compensation theory lies in: First of all, teachers should actively guide students to establish correct and appropriate compensation goals, on this basis, to carry out frustration education and life education for students, cultivating students’ awareness of compensation, and teaching them to face up to their own shortcomings with appropriate methods; secondly, on the hand of students, they should correctly treat their own shortcomings and the advantages of others, on this basis, to establish the correct life values to exceed the present themselves; finally, society should also give students more care, encouragement, support and help so that they can get compensation in society, so as to improve their self-confidence.

## Conclusions

Due to the difference in self-concept in the context of Eastern and Western cultures, Western individualism does not include anyone else, most advocate personal values and habitually distinguish themselves from the outside world; The Eastern collectivist self includes the perception of others, so in a cross-cultural context, compensatory behavior is not necessarily done by the inferior self, but can also be done by others or groups. For example, a study by Neumann [45] noted that Chinese subjects felt more proud of the successes of others than German subjects, suggesting that individuals in collectivist cultures may be more likely to be compensated by others than individuals in individualistic cultures. In other words, the inferior individual in the context of collectivist culture does not regard the honor of others as a threat, but takes the initiative to actively view the success of others, and feels the strength from others to motivate themselves, which can also be said to be the so-called inferiority compensation in Adler’s theory.

1. Adler’s theory of inferiority compensation can exist in a cross-cultural context in ways of others compensation.
2. The compensative model of inferiority was confirmed, and the inferiority-compensation scale duly included two subscales: self-inferiority-compensation and other-inferiority-compensation.
3. As a reliable and valid measure, this scale can be used to measure the way of inferiority-compensation among high school students.

## Supplementary Information

**Additional file 1. Appendix 1.** Inferiority compensation questionnaire for high school students (formal questionnaire).

## Data Availability

Data associated with this article be contacted (or if someone wants to request the data from this study) can be found in the online version at https://pan.baidu.com/s/1nMAlZ1ZZf-QPYT_DFjn_7gcode:27 × 9 and authors Dr. Yang can be contacted.
