# Supplementary material for: Development of inferiority-compensation scale among high school students
Source: BMC Med Educ. 2023 Jan 12;23:23. doi: 10.1186/s12909-022-03979-3 (PMC9837908; doi:10.1186/s12909-022-03979-3)
Supplement: Supplementary file 1 — Additional file 1. Appendix 1. Inferiority compensation questionnaire for high school students (formal questionnaire). [file 12909_2022_3979_MOESM1_ESM.docx]

**Development of inferiority-compensation scale among high school students**

**Di Yang^1, 2, #^, Baiyang Qiu^1,2#^, Jin Jiang^1,2^, Youkui Xia****^1,2^, Lingxiao Li^1,2^, Yanting Li^1,2^, Longli Luo^1,2^, Xiaocui Liu^1,2^ and Jing Meng^1,2^**

^1^ Key Laboratory of Applied Psychology, Chongqing Normal University, Chongqing, China

^2^ School of Education, Chongqing Normal University, Chongqing, China

**APPENDIX**

Appendix 1：Inferiority compensation questionnaire for high school students（formal questionnaire）

| Item | | Completely inconsistent | Basically inconsistent | Uncertain | Basically consistent | Completely consistent |
| --- | --- | --- | --- | --- | --- | --- |
| 1 | Because one subject is poor, I will make up for it by taking advantage of other subjects | 1 | 2 | 3 | 4 | 5 |
| 2 | Because of the weak reading ability, I will make up by reading extracurricular books | 1 | 2 | 3 | 4 | 5 |
| 3 | Because a subject is not good enough, I will strive to study this weak subject. | 1 | 2 | 3 | 4 | 5 |
| 4 | Because of my timidity, I will speak actively in class. | 1 | 2 | 3 | 4 | 5 |
| 5 | Because I don 't want to admit I got poor grades, I ' ll brag about myself in front of my friends and relatives. | 1 | 2 | 3 | 4 | 5 |
| 6 | Because of the weak sports scores, I make up for the promotion by attending sports meetings | 1 | 2 | 3 | 4 | 5 |
| 7 | Because of poor physical coordination, I often practice yoga to make up for it. | 1 | 2 | 3 | 4 | 5 |
| 8 | Because of the poor grades in the general knowledge courses, I will try to exercise sports skills to make up for this deficiency. | 1 | 2 | 3 | 4 | 5 |
| 9 | Because of the weak language expressing ability, I usually take the initiative in communicating with others | 1 | 2 | 3 | 4 | 5 |
| 10 | Because of the weak communication skills, I often take the initial in making friend with others | 1 | 2 | 3 | 4 | 5 |
| 11 | Because of my poor talent and skill, I often take an active part in various club activities | 1 | 2 | 3 | 4 | 5 |
| 12 | I praise myself in front of the opposite sex to be looked up to | 1 | 2 | 3 | 4 | 5 |
| 13 | I don’t want my parents to know I have no friends, so I tried hard to hide it | 1 | 2 | 3 | 4 | 5 |
| 14 | Because the skin is swarthy, I often wear some light color clothes to hide | 1 | 2 | 3 | 4 | 5 |
| 15 | I don't want to admit I'm fat, so I lie about my weight | 1 | 2 | 3 | 4 | 5 |
| 16 | Because my shape is not good enough, I often wear some clothes with vertical stripes to hide | 1 | 2 | 3 | 4 | 5 |
| 17 | I read self-help books to motivate myself | 1 | 2 | 3 | 4 | 5 |
| 18 | In order to make myself sensible, I will take the initiative to help my parents undertake the housework chores | 1 | 2 | 3 | 4 | 5 |
| 19 | In order to have a kind heart, I often offer to help others | 1 | 2 | 3 | 4 | 5 |
| 20 | In order to make myself graceful, I will read a lot of books to improve myself | 1 | 2 | 3 | 4 | 5 |
| 21 | I don't like to admit that I'm incompetent, so I often brag about myself | 1 | 2 | 3 | 4 | 5 |
| 22 | The development of the school makes me full of confidence in my future study | 1 | 2 | 3 | 4 | 5 |
| 23 | A relative's child was admitted to a selective school through hard work, which indirectly increased my confidence in the future | 1 | 2 | 3 | 4 | 5 |
| 24 | I often ignore teachers' compliments on my friends' work | 1 | 2 | 3 | 4 | 5 |
| 25 | A certain subject teacher participated in the competition and won an honorary award, which made me more confident in learning this subject | 1 | 2 | 3 | 4 | 5 |
| 26 | A very over-weight friend lost weight and became handsome, which convinced me that I could lose weight, too | 1 | 2 | 3 | 4 | 5 |
| 27 | My friend with poor physical fitness participated in the sports meeting and won an honorary award, which made me believe that my physical fitness is not bad | 1 | 2 | 3 | 4 | 5 |
| 28 | I often ignore the P.E. teacher's praise of my friend for an excellent sport score | 1 | 2 | 3 | 4 | 5 |
| 29 | Timid friends took the initiative to ask the teacher for advice on study and got praise, which increased my courage to ask the teacher for advice | 1 | 2 | 3 | 4 | 5 |
| 30 | My parents always get on well with their neighbors, which give me confidence in my own ability to deal with people | 1 | 2 | 3 | 4 | 5 |
| 31 | Good friends being popular in class makes up for my disappointment at not having any friends | 1 | 2 | 3 | 4 | 5 |
| 32 | My desk-mate has a good relationship with the students in the next class, which makes me believe that I can also make friends with the next class | 1 | 2 | 3 | 4 | 5 |
| 33 | I often ignore the teacher's praise on my desk-mates well-connections | 1 | 2 | 3 | 4 | 5 |
| 34 | My parents chat well with other parents at parent-teacher conferences, which make me believe I can relate to their kids, too | 1 | 2 | 3 | 4 | 5 |
| 35 | I often overlook the fact that my homely classmates are praised by the teacher for their efforts | 1 | 2 | 3 | 4 | 5 |
| 36 | The homely relative starts a relationship, which indirectly convinces me that I am not so bad-looking | 1 | 2 | 3 | 4 | 5 |
| 37 | Physically disabled teachers stand on the platform by their own efforts, which makes me believe that appearance is not the most important thing | 1 | 2 | 3 | 4 | 5 |
| 38 | The country's prosperity fills me with confidence in the future development of society | 1 | 2 | 3 | 4 | 5 |
| 39 | My parents' work becomes better and better, which makes me more confident in front of my classmates | 1 | 2 | 3 | 4 | 5 |
| 40 | The growth of the class convinces me that I am not incompetent | 1 | 2 | 3 | 4 | 5 |
| 41 | I often overlook my friend's success through hard work | 1 | 2 | 3 | 4 | 5 |

Note: 1-21 constitute the self-compensation sub-scales; 22-41 constitute the sub-scale of others-compensation. 1-5, 6-8, 9-13, 14-16, 17-21; 22-25, 26-28, 29-34, 35-37, 38-41 are academic performance, physical fitness, social communication, appearance, self-esteem respectively. (5, 13, 21, 24, 28, 33, 35, 41 are reverse scoring questions).
